# Exploring the differences between sole silages of gramineous forages and mixed silages with forage legumes using 16S/ITS full-length sequencing

**DOI:** 10.3389/fmicb.2023.1120027

**Published:** 2023-03-02

**Authors:** Xianjun Lai, Haiyan Wang, Junfeng Yan, Yizheng Zhang, Lang Yan

**Affiliations:** ^1^Panxi Crops Research and Utilization Key Laboratory of Sichuan Province, College of Agriculture Science, Xichang University, Liangshan, China; ^2^Sichuan Key Laboratory of Molecular Biology and Biotechnology, College of Life Sciences, Sichuan University, Chengdu, China; ^3^Chengdu Ke’an Technology Co., Ltd., Chengdu, China; ^4^Mianyang Youxian Innovation Technology and Industrial Technology Research Institute, Mianyang, China

**Keywords:** gramineous grass, forage legume, mixed silage, fermentation quality, bacterial and fungal community

## Abstract

**Background/Objective:**

Silage characteristics of grass materials directly affect their silage qualities. To expand the source of silage raw materials and develop mixed silages underlined by exploring the positive interactions between forage grasses and legumes, three gramineous grasses, Napier grass (*Pennisetum purpureum*), king grass (*Pennisetum sinese*), and forage maize (*Zea mays*) were separately mixed ensiled with a combination of four forage legumes including *Medicago sativa*, *Vicia villosa*, *Vicia sativa*, and *Trifolium repens*.

**Methods:**

The chemical composition and fermentation quality of the mixed silages were analyzed and compared with those of the sole silages of these three grasses, as well as the diversity of microbial communities, through the 16S/ITS full-length sequencing.

**Results:**

The results showed that the inclusion of forage legumes could somewhat improve the fermentation quality, as indicated by significantly (*p* < 0.05) higher crude protein and lactic acid contents while lower neutral detergent fiber, acid detergent fiber contents and pH values, compared with the sole silages. Among the three types of mixed silages, the mixed king grass had the highest dry matter and crude protein content as well as lowest neutral detergent fiber and acid detergent fiber content. Meanwhile, the bacterial and fungal communities in the mixed silages were influenced by increased the relative abundance of lactic acid bacteria, which inhibited the proliferation of undesirable bacteria, such as *Hafnia alvei*, *Enterobacter cloacae*, and *Serratia proteamaculanss*. Co-occurrence networks identified 32 nodes with 164 positive and 18 negative correlations in bacteria and 80 nodes with two negative and 76 positive correlations in fungi during fermentation.

**Conclusion:**

Inclusion of forage legume to grasses can improve the fermentation quality and optimize the structure of microbial community, which appears to be a feasible strategy to enhance the forage resource utilization.

## Introduction

High-quality forage accounts for ~70% of the cost of ruminant rearing and is important for the development of animal husbandry (Hisham et al., 2022). The continental plateau climate of the mountainous areas of southwest China experiences seasonal changes, which leads to the depletion of forage resources in winter and spring, shortage of coarse fodder for cattle and sheep, and fat loss and even death of livestock (Li X. et al., 2015). In the absence of fresh plant feed, silage can preserve the fresh grass for a long time, reduce nutrient loss, and can be better digested and absorbed by the animals (Kung et al., 2018; Liu et al., 2022). Silage of excess forage is of great significance in contributing to the seasonal supply of livestock feed, thereby ensuring that ruminants can survive the winter. Moreover, breeders can benefit from replacing expensive imported feed with silage for cheaper livestock production (Kung et al., 2018; Hisham et al., 2022).

Ensiling involves microbial fermentation, which transforms sugars into acids and reduces the pH under anaerobic conditions, and ultimately, inhibits the proliferation of undesirable microorganisms (Fabiszewska et al., 2019). The success of ensiling is affected by various factors that can be roughly divided into two categories: forage features and silage conditions (McEniry et al., 2008; Zhang et al., 2016). The epiphytic microbial community of forage materials or the use of exogenous inoculants play a vital role, and external factors, such as climatic conditions, silage facilities, and harvesting periods, provide excellent conditions for microbial fermentation (Kung et al., 2018). For example, a rapid decline in pH during the early stages of fermentation, which is a key determinant of silage quality, can inhibit spoilage microorganisms from degrading proteins and produce NH_3_–N (Ouamba et al., 2022). Because fermentation quality is directly related to the microbial community, studies on bacterial and fungal communities during ensiling not only perceived the principle of ensiling but also established the role of the key microorganisms during fermentation (McEniry et al., 2008; Naoki and Yuji, 2008).

Different forage materials have different silage characteristics, which directly affect silage quality. Gramineous forages, such as corn (*Zea mays* L.), sorghum (*Sorghum bicolor* L.), Napier grass (*Pennisetum purpureum* Schum.), and hybrid giant Napier (*Pennisetum sinese* Roxb., namely king grass), are C4 grasses belonging to poaceae and kinds of ideal forage type, because of its high biomass yield, sufficient substrate for the fermentation of sugars and low buffering capacity, which contributes to reduce pH quickly below the general standard of 4.2 in silage fermentation (Zhang et al., 2011; Li et al., 2014; Santos et al., 2016; Alhaag et al., 2019). Napier grass and king grass, originated from tropical regions, were introduced and cultivated in the mountainous areas of southwest China in the 1990s and have been domesticated to adapt to the local habitat. Although the stems and leaves of grass forages are ideal for developing ruminant feed, involving in reducing the feed cost and enriching roughage resources, providing only silages of grass does not satisfy the nutritional requirements of highly producing ruminants because of its high fiber content, low digestibility and protein content, which limited their application in ruminant feed production (Zi et al., 2021). Compared to gramineous silages, legume silages typically increase dry matter (DM) intake and performance of ruminant production (Dewhurst et al., 2009). Forage legume, such as alfalfa (*Medicago sativa* L.), white clover (*Trifolium repens* L.) and common vetch (*Vicia sativa* L.), which provided a good source of crude protein (CP) and crude fat (*CF*), are high-quality, unconventional feed resources supporting high DM intake even with high-producing dairy cows fed high-forage diets (Wüstholz et al., 2017; Zheng et al., 2019). However, high proportions of legumes may enhance silage nutritive value but may also reduce silage quality (Wang et al., 2017). Forage legumes are lower in neutral detergent fiber (NDF) and sugar than gramineous forages, which contributes to their high buffering capacity and makes them more difficult to ensile than grass species under natural fermentation conditions (Filya et al., 2007; Ni et al., 2018). Therefore, if forage legumes could substitute a part of grass forage and are conserved well as mixed silage, it would enlarge the feed resource, stabilize the fermentation system and enhance the silage resource utilization.

In recent years, many researches had compared a range of legume-cereal combinations in terms of fermentation characteristics, nutritive quality and *in vitro* digestibility, and have shown that the ratio of legume to grass or cereal had evident effects on fermentation patterns and resultant silage nutrition (Zeng et al., 2020; Li et al., 2022; Wang et al., 2022). According to a report, compared with sole silage, mixed silage not only increased lactic acid (LA) production and reduced pH, but reduced the production of propionic acid and ammonia nitrogen (Kung et al., 2018). Wang et al. elaborated that the inclusion of legumes could somewhat improve the corn stover silage quality with higher ratios of LA/acetic acid (AA), and *in vitro* DM and NDF digestibility (Wang et al., 2017). They also showed that inclusion of alfalfa to mixtures of straws and tall fescue had favorable effects on fermentation quality and obviously improved the nutritive value and *in vitro* digestibility of mixed silages (Wang et al., 2018). Given this, it is necessary to investigate the effect of forage legumes mixed with tall gramineous grass silages, especially the differences in microbial composition between sole and mixed silages. To the best of our knowledge, scant information is available on the strategy of preservation and utilization of tropical originated tall gramineous grass silage applied in the temperate mountain area.

The purpose of this study was to evaluate the effect of ensiling of three types of gramineous grasses (Napier grass, king grass, and forage maize) with forage legumes in mixed silage on fermentation characteristics, nutritive quality and microbial community diversity. Identifying the connection between silage quality and microbial community would provide a theoretical basis for developing mixed silages of gramineous and legumes forage.

## Materials and methods

### Materials and silage preparation

All plants were planted and harvested at the experimental field of Xichang University (Anning town, Liangshan, China: 30°34′N, 104°4′ E, elevation 1,574 m). The stems, nodes, and leaves of three gramineous grasses: *P. sinese*, called king grass; *P. purpureum*, called Napier grass; and *Z. mays*, called forage maize, ~60-day-old and 1.2 ~ 1.5 m in height, were harvest at stubble height at 10 ~ 12 cm. Mowed forages were chopped into theoretical lengths of 10 ~ 20 mm using a high-speed chopper. In addition, four leguminous forages, including alfalfa (*M. sativa*), white clover (*T. repens*), hairy vetch (*Vicia villosa* Roth.) and common vetch (*V. sativa*) were harvested and combined in equal proportions as additive. Alfalfa was mechanically harvested at the early-bloom stage from the second cutting, leaving a 5 cm stubble and then chopped to a length of 20 mm with a forage cutter. White clover was harvested for ensiling at the early-flowering stage following 8 weeks of growth. Plant material was chopped by hand using scissors to lengths of approximately 10 ~ 20 mm. Hairy vetch and common vetch were harvested with a 5 cm stubble height. Hairy vetch was at the early podding period and common vetch was at the bloom period.

All silage treatments based on fresh matter are as follows: (1) 100% king grass; (2) 100% forage maize; (3) 100% Napier grass; (4) combination of 70% king grass and 30% leguminous forage; (5) combination of 70% forage maize and 30% leguminous forage; and (6) combination of 70% Napier grass and 30% leguminous forage. The adding proportion (30%) of forage legume was according to the results of the early preliminary test (data not shown). Each sample of 1 kg, in triplicate, was packed into polyethylene plastic bags (dimensions 25 cm × 35 cm), and then vacuum sealed. A total of 18 bags were preserved at room temperature. The chemical composition, fermentation quality, and microbiota community were analyzed for samples after 60 days of ensiling.

### Measurement of chemical compositions and fermentation quality

Fresh and ensiled samples were analyzed for DM, water-soluble carbohydrate (WSC), CP, ether extract (EE), *CF*, NDF, and acid detergent fiber (ADF). DM contents of samples were determined using the 934.01 AOAC method (2016) by drying in an oven at 115°C until the weight becomes constant. CP and EE were determined according to the Guidelines of the Association of Official Analytical Chemists (AOAC, 2005). NDF and ADF were determined as described (He et al., 2020). WSC was determined in accordance with the methods described by Dubois et al. CP measurement was conducted using the Kjeldahl method, Latimer (2016).

The fermentation quality of silage was determined using distilled water extracts. Briefly, 50 g wet silage was homogenized with 180 ml sterilized water for 1 min in a blender, incubated at 4°C for 24 h, and filtered through medical gauze with four layers. The pH of filtrate was detected with a pH meter (PHSJ-5; LEICI, Shanghai, China). LA and AA were determined by high-performance liquid chromatography (Agilent 1,100, United States), as described (Zeng et al., 2020).

### Microbial diversity analysis

The E.Z.N.A. Soil DNA Kit (Omega Bio-Tek, United States) was used to isolate microbial DNA from silage samples according to the instructions of the manufacturer. DNA concentration ≥ 20 ng/ml, detected using the ultra-micro spectrophotometer (NanoDrop 2000), was qualified if OD260/280 = 1.8–2.0 and OD260/230 > 2.0.

Total DNA extracted was diluted to 3.5 ng/μL and stored at −20°C for PCR amplification. Full-length bacterial 16S rRNA and fungal ITS regions were amplified for PacBio full-length sequencing. Briefly, a specific barcoded primer was synthesized according to the full-length primer sequence. The full-length of 16S rRNA gene and ITS region were amplified using a thermocycler. Then the product was purified, quantified, and homogenized to build a sequencing library (SMRT Bell). The quality of the library was inspected and the library was sequenced using the PacBio Sequel system. To deal with the BAM format of the PacBio Sequel offline data, the Smrtlink software was used to export CCS files, data from samples were identified by the barcode sequence, and converted into data in the FastQ format.

For data preprocessing, the Lima v1.7.0 software^1^ was used to identify circular consensus sequencing (CCS) reads through the barcode and obtain raw CCS sequences. Cutadapt 1.9.1 (Martin, 2011) was used to identify and remove primer sequences and obtain clean CCS sequences without primer sequences through sequence length filtering. UCHIME v4.2 (Edgar et al., 2011) was used to identify and remove chimeric sequences and obtain effective CCS sequences. The high-quality sequences were clustered into OTUs defined at a similarity of 97%. The core-diversity plug-in within QIIME2 (Hall and Beiko, 2018) was used to determine diversity metrics. Microbial diversity within an individual sample was assessed using the α-diversity indices, including observed OTUs, Chao1 richness estimator, Shannon’s diversity index, and Simpson and ACE indices. *β*-diversity was analyzed to assess the structural variation of microbiota across specimens, and then PCoA was performed. Linear discriminant analysis (LDA) effect size (LEfSe) was performed using the LEfSe software. Spearman’s rank correlation analysis was performed and the data with correlation greater than 0.6 and *p*-value <0.05 were selected to construct the correlation network. The coexistence relationship of a species in environmental samples was obtained based on the analysis of network graphs. The sequencing data were deposited in the Sequence Read Archive (SRA) under the accession number PRJNA909078 for 16S rRNA sequencing and PRJNA909075 for ITS sequencing.

### Statistical analysis

The impacts of forage species, silage methods, and their interactions were investigated by two-way factorial ANOVA using the aov() function in R language. Significant differences of the least significant difference (LSD) tests were conducted using agricolae package in R, and *p* < 0.05 was considered statistically significant. Microbial enumeration data were log-transformed prior to statistical analysis. For *α*-diversity, significant differences between groups were calculated based on ANOVA or *t*-test at *p*-value <0.05. Beta diversity was calculated on the basis of the Bray–Curtis distance, and statistical comparisons between groups were performed using PERMANOVA. LEfSe analysis was conducted at the feature level with LDA score > 4.0 capped at FDR-adjusted *p*-value <0.05.

## Results

### Characteristics of raw materials and fermentation quality after ensiling

The chemical composition of each raw material is shown in Table 1. The DM content of fresh king grass was 28.97%, which was much higher than that of forage legumes, such as alfalfa (22.07%), common vetch (18.87%), and white clover (11.46%). Moreover, king grass had the highest WSC content (183.00 g/kg of DM), which was significantly higher than that of forage legumes, ranging from 33.35 to 91.70 g/kg of DM. Forage legumes represented by alfalfa had a relatively high CP content (18.10% of DM), whereas king grass, Napier grass, and forage maize had a CP content of 8.26, 4.33, and 6.67 g/kg DM, respectively. Poaceae grasses were relatively rich in *CF*, with the contents ranging from 21.10 to 30.90% of DM. Although the values of NDF content between poaceae and forage legumes were comparable, the ADF content of poaceae grasses, especially king grass (37.25% of DM) and Napier grass (40.55% of DM), was significantly higher than that of forage legumes.

**Table 1:** Chemical composition in fresh raw materials.

Item	Alfalfa	Hairy vetch	White clover	Common vetch	King grass	Forage maize	Napier grass
Dry matter (% FM)	22.07 ± 0.28^c^	20.25 ± 0.06^d^	11.46 ± 0.15^f^	18.87 ± 0.52^e^	28.97 ± 0.43^a^	24.18 ± 0.20^b^	20.13 ± 0.24^d^
WSC (g/kg of DM)	79.65 ± 10.65^c^	91.70 ± 20.40^c^	50.10 ± 19.90^d^	33.35 ± 12.55^d^	183.00 ± 11.40^a^	125.40 ± 4.10^b^	179.30 ± 11.70^a^
CP (% DM)	18.10 ± 0.03^a^	13.58 ± 0.00^d^	14.34 ± 0.15^c^	15.76 ± 0.25^b^	8.26 ± 0.10^e^	6.67 ± 0.00^f^	4.33 ± 0.17^g^
EE (% DM)	2.85 ± 0.05^f^	3.05 ± 0.05^e^	3.60 ± 0.00^b^	3.70 ± 0.00^a^	3.35 ± 0.05^c^	2.30 ± 0.00^g^	3.25 ± 0.05^d^
CF (% DM)	17.90 ± 0.20^e^	23.65 ± 1.15^c^	16.75 ± 0.25^f^	14.15 ± 0.15^g^	30.90 ± 0.60^a^	21.10 ± 0.10^d^	28.55 ± 0.65^b^
NDF (% DM)	75.05 ± 0.85^a^	67.85 ± 0.95^c^	68.95 ± 0.25^c^	70.80 ± 0.70^b^	67.90 ± 0.90^c^	71.75 ± 0.55^b^	66.30 ± 0.50^d^
ADF (% DM)	21.85 ± 0.35^f^	32.55 ± 0.55^c^	26.55 ± 0.25^e^	32.85 ± 0.25^c^	37.25 ± 0.05^b^	30.40 ± 0.40^d^	40.55 ± 0.25^a^

FM, fresh matter; DM, dry matter; WSC, water-soluble carbohydrate; CP, crude protein; EE, ether extract; CF, crude fiber; NDF, neutral detergent fiber; ADF, acid detergent fiber. a–g means values within the same row with different superscripts in lowercase letters differ significantly from each other.

The same indicators of chemical composition as well as fermentation quality after 60 days of sole and mixed ensiling were presented in Table 2. The DM content of the three grasses decreased after 60 days of ensiling. Notably, the interactions between forage species and silage methods influenced DM content. The addition of forage legumes to mixed silages significantly decreased DM content compared with the sole silages of the three grasses individually. However, mixed silages had higher CP content and lower NDF and ADF content than sole silages, suggesting that mixed silage facilitates animal digestion and absorption. Between the three types of mixed silages, the chemical compositions of king grass were better than that of the other two grasses, which had higher DM and CP content as well as lower NDF and ADF content. Moreover, fermentation quality was affected by grass species in that mixed silages had lower pH value and higher LA content than sole silages. Although the concentration of acetic acid (AA) was significantly higher in mixed silages than sole silages due to lower DM content, the pH of both king grass and forage maize mixed silages significantly decreased to <4 after 60 days, which demonstrating the better fermentation quality of mixed silages.

**Table 2:** Chemical composition and fermentation quality after ensiling.

	Sole silage			Mixed silage			SEM	*P*-value			King grass	Forage maize	Napier grass	King grass	Forage maize	Napier grass	*F*	*S*	F*S
DM (% FM)	27.05 ± 0.85^a^	22.85 ± 0.95^c^	18.95 ± 0.25^e^	24.80 ± 0.70^b^	20.90 ± 0.90^d^	18.75 ± 0.55^e^	0.75	**	**	*
CP (% DM)	14.39 ± 0.03^b^	10.26 ± 0.13^e^	8.67 ± 0.01^f^	16.54 ± 0.11^a^	11.36 ± 0.13^c^	10.87 ± 0.12^d^	0.64	**	**	**
NDF (% DM)	57.30 ± 0.80^c^	62.55 ± 1.15^a^	59.15 ± 0.75^b^	51.55 ± 1.35^d^	60.45 ± 1.15^b^	51.80 ± 0.40^d^	1.03	**	**	**
ADF (% DM)	28.00 ± 0.40^c^	29.35 ± 0.25^b^	33.20 ± 0.20^a^	23.35 ± 0.25^e^	25.70 ± 0.20^d^	27.80 ± 0.30^c^	0.74	**	**	**
pH	4.15 ± 0.01^b^	4.05 ± 0.01^c^	4.21 ± 0.00^a^	3.85 ± 0.00^d^	3.85 ± 0.01^d^	4.05 ± 0.00^c^	0.03	**	**	**
LA (% DM)	2.79 ± 0.20^b^	2.65 ± 0.15^b^	2.75 ± 0.25^b^	3.15 ± 0.15^a^	3.09 ± 0.10^a^	3.10 ± 0.10^a^	0.06	NS	**	NS
AA (% DM)	0.85 ± 0.05^c^	0.95 ± 0.06^bc^	1.07 ± 0.05^b^	1.25 ± 0.15^a^	1.27 ± 0.07^a^	1.40 ± 0.10^a^	0.05	**	**	NS

FM, fresh matter; DM, dry matter; CP, crude protein; EE, ether extract; CF, crude fiber; NDF, neutral detergent fiber; ADF, acid detergent fiber. F, forage species; S, silage methods; F*S, the interaction between forage species and silage methods; SEM, standard error of the mean; a–f means values within the same row with different superscripts in lowercase letters differ significantly from each other at *P* < 0.05; * and **, significant at *P* < 0.05 and 0.01, respectively; NS, not significant.

### Bacterial communities in sole and mixed silages

**Figure 1:**
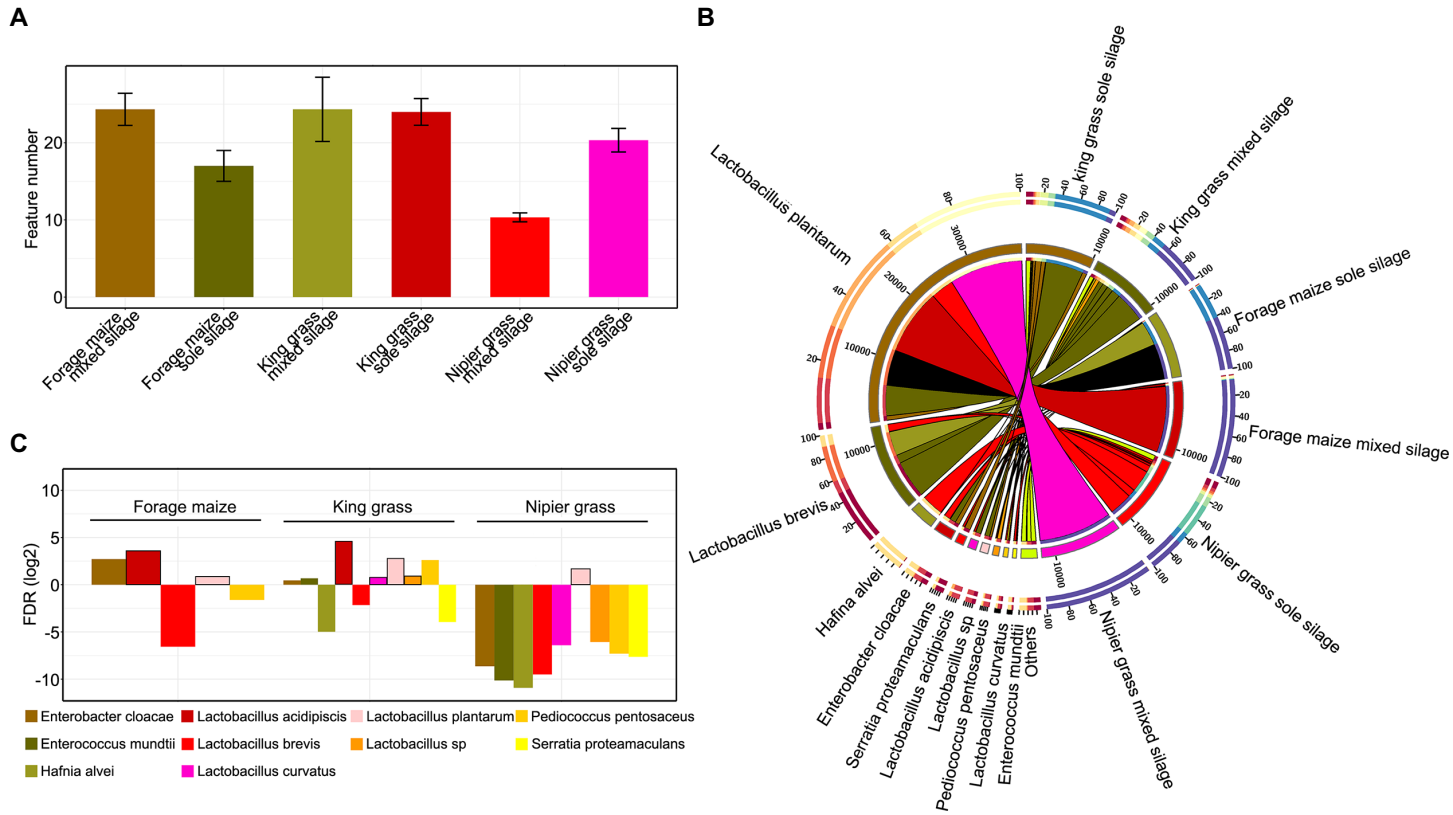
Patterns of relative abundance of bacterial communities between samples. **(A)** Feature numbers of operational taxonomic units (OTUs) in each sample. **(B)** Distribution of the 10 most abundant bacterial species of silages. The bar length on the outer ring represents the percentage of each species in each sample. **(C)** The ratio of log2 fold change in the relative abundance of bacterial taxa in mixed silages compared with sole silages as control. The quantity of OTUs was normalized to unity.

A total of 233,518 CCS sequences were obtained from 18 samples by full-length 16S rRNA sequencing (PacBio-SMRT Cell). After length filtering and chimera removal, 11,025–12,097 effective CCS sequences were generated in each sample. These were clustered into 52 operational taxonomic units (OTUs) at 97% sequence similarity, represented by different bacterial species in the samples. Due to the relatively closed fermentation environment, fewer OTU species were present in the sample (10–29), and OTU numbers were relatively higher in mixed silages than in sole silages of king grass and forage maize. However, for Napier grass, the mixed silage had fewer bacterial OTU species than the sole silage, with approximately one-half reduction (Figure 1A). The percentage abundance of bacterial species showed significant differences of the dominant bacterial species between samples (Figure 1B). *Lactobacillus plantarum*, *Lactobacillus brevis*, *Hafnia alvei*, *Enterobacter cloacae*, and *Serratia proteamaculans* were the top five bacterial species that dominated the bacterial community of silages. *L. brevis* was dominant in king grass and forage maize in sole silages. However, the abundance of *L. brevis* decreased sharply when they were fermented mixed with forage legumes. In mixed silages, *L. plantarum* was dominant after ensiling, especially in forage maize and Napier grass. Other bacterial species, such as *H. alvei*, *E. cloacae*, and *S. proteamaculans*, which are undesirable for silage fermentation, were detected in sole silages of king and Napier grasses but decreased sharply in mixed silages (almost disappeared in Napier grass) (Figure 1C).

**Figure 2:**
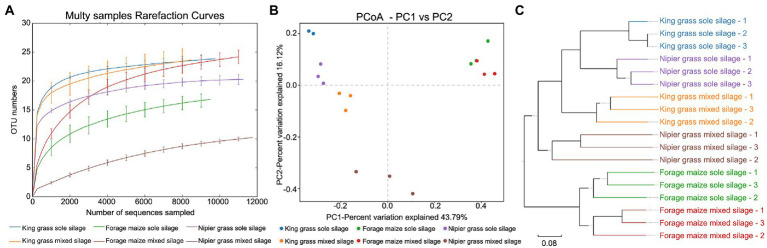
Diversity of bacterial communities between sole and mixed silages. **(A)** Rarefaction curves for *α*-diversity measures of operational taxonomic units (OTUs) comparing bacteria from sole and mixed silages. Error bars correspond to one standard deviation from the average (*n* = 3 biological replicates). **(B)** Unconstrained principal coordinates analysis (PCoA) (for principal coordinates PCo1 and PCo2) with weighted UniFrac distance showing that bacteria in different samples separate in the first axis (*p* < 0.001, PERMANOVA). **(C)** Hierarchical clustering trees in unweighted pairwise average method (UPGMA) analysis. Distance matrices were obtained using the weighted UniFrac distance algorithm. The samples that are closer (with shorter branch length) share higher similarity in species compositions.

Measurement of within-sample diversity (*α*-diversity) revealed significant differences between grass materials as well as ensiling method. The rarefaction curve showed that all samples reached a plateau, indicating that the sequencing had adequately captured most of the bacterial community. Importantly, rarefaction plots of OTUs, Faith’s PD, and Shannon’s index (*t*-test, *p* < 0.05, data not shown) confirmed the presence of a significant correlation between bacterial community diversity and grass material (Figure 2A). The microbiota from silages of king grass was significantly more diverse than that of forage maize and Napier grass in both sole and mixed silages. Regarding community diversity, forage maize exhibited lower species richness and diversity in sole silages and higher diversity in mixed silages than Napier grass, indicating that the silage of forage maize mixed with forage legumes resulted in the recruitment of more bacterial species. By contrast, Napier grass mixed with forage legumes significantly decreased the abundance and diversity of the bacterial communities.

To evaluate the degree by which bacterial communities were influenced by the grass material and silage method and factor(s) regulating bacterial community variation, principal coordinates analysis (PCoA) was conducted based on the weighted UniFrac distance matrix in combination with PERMANOVA (Figure 2B). PCoA revealed that the microbiota of silages formed three distinct clusters, which separated along the first coordinate. Silages of forage maize were separated from those of the other two grasses, indicating that the largest source of variation in fermentative microbiota of forage maize was proximity to the grass species rather than the ensiling method. However, the sole and mixed silage of king grass and Napier grass are completely separated on the first coordinate, and are clustered according to the silage method along with the second coordinate. Based on the four distance matrices obtained by *β*-diversity analysis, the samples were hierarchically clustered using the unweighted pairwise average (UPGMA) method to evaluate similarity of species composition between samples. King grass and Napier grass had similar bacterial community structures in both sole and mixed silages, which were separated from that in silages of forage maize (Figure 2C). This suggests that the composition of the fermentative bacterial community in king grass and Napier grass is mainly affected by the ensiling method and marginally influenced by the grass material.

### Fungal communities of silages

**Figure 3:**
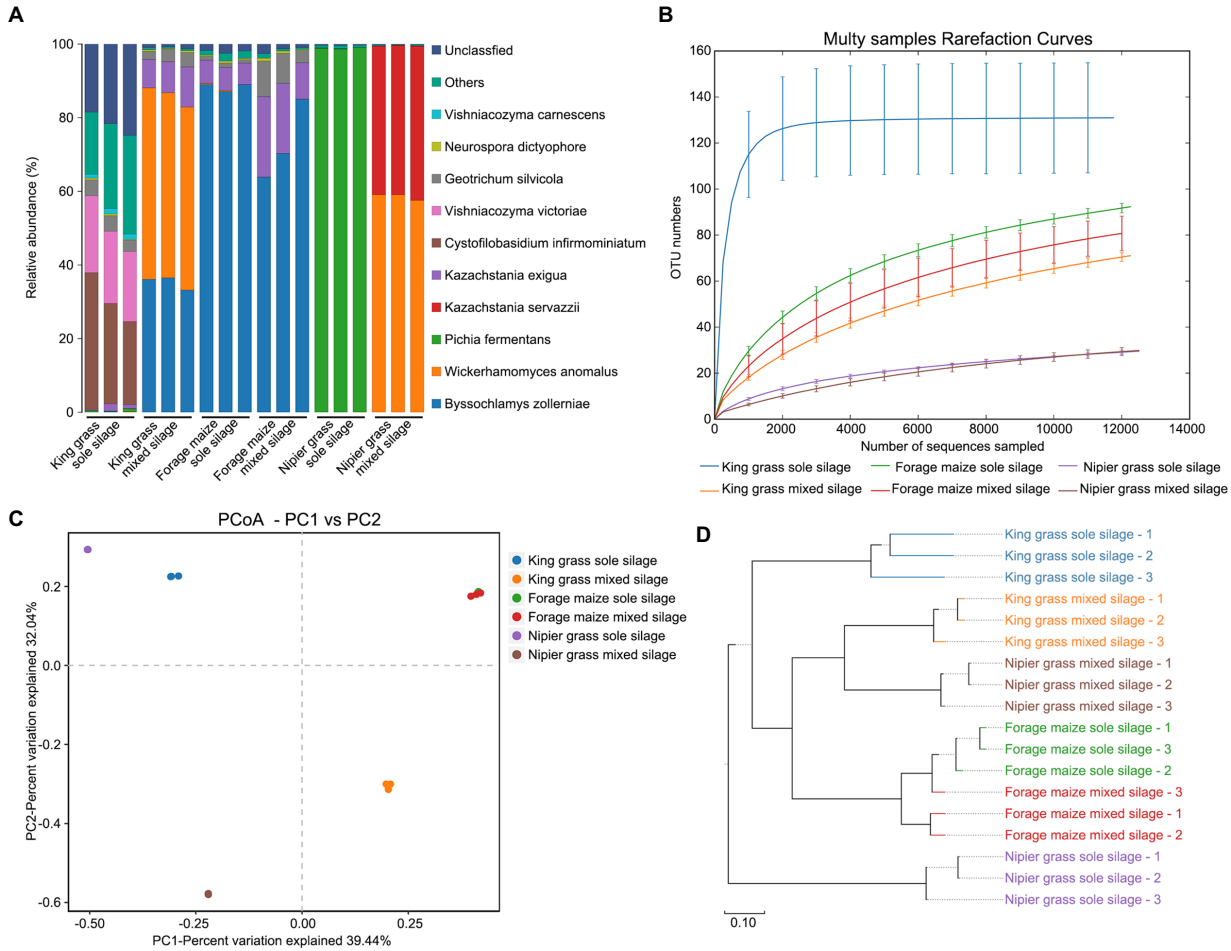
Diversity of fungal communities between sole and mixed silages. **(A)** Relative abundance of bacteria at the species level. **(B)** Rarefaction curves for *α*-diversity measures of operational taxonomic units (OTUs) comparing fungal communities from sole and mixed silages. Error bars correspond to one standard deviation from the average (*n* = 3 biological replicates). **(C)** Unconstrained principal coordinates analysis (PCoA) (for principal coordinates PCo1 and PCo2) with weighted UniFrac distance showing that fungal communities in different samples separate in the first axis (*p* < 0.001, PERMANOVA). **(D)** Hierarchical clustering trees in unweighted pairwise average method (UPGMA) analysis. Distance matrices were obtained by the weighted UniFrac distance algorithm. The samples that are closer (with shorter branch length) share higher similarity in species composition.

The fungal communities of the six silage types were identified through the full-length sequencing of the ITS1 region. A total of 227,341 effective CCS sequences, with an average of 12,630 sequences per sample, were clustered in 324 OTUs at 97% sequence similarity. Quantitatively, sole silages had larger quantities of fungi than mixed silages, especially in king grasses, accounting for one-third of the fungal population of the silages, with 135 specific OTUs. The percentage abundance of fungal species is shown in Figure 3A. Significant differences of the dominant fungal species between sole and mixed silages, especially in king grass and Napier grass, were observed. In the sole silage of king grass, the fungal communities comprised mildew and yeasts, including *Cystofilobasidium infirmominiatum*, *Vishniacozyma victoriae*, and *Geotrichum silvicola*, which are undesirable for improving silage quality. However, fungi such as *Wickerhamomyces anomalus*, *Byssochlamys zollerniae*, and *Kazachstania exigua* dominated the fungal community in the mixed silage of king grass. Similarly, *Pichia fermentans* which dominated the sole silage of Napier grass, was completely replaced by *W. anomalus* and *Kazachstania servazzii* when the sole silage was fermented with forage legumes. The types and quantities of fungal communities were almost consistent between sole and mixed silages of forage maize.

The rarefaction curve showed the *α*-diversity of fungal community diversity within samples (Figure 3B). Notably, microbial communities from sole silages were significantly more diverse than those from mixed silages. The sole silage of king grass had the highest diversity, followed by the sole silages of forage maize and Napier grass. The mixed silage of king grass and forage legumes had a significant decrease in the abundance and diversity of fungal communities. PCoA, conducted based on the weighted UniFrac distance matrix in combination with PERMANOVA, revealed that the microbiota of silages separated along the first coordinate (Figure 3C). Sole and mixed silages of forage maize were clustered together, whereas those of king grass and Napier grass were separated in both sole and mixed silages. Thus, ensiling method affects the fungal communities of king grass and Napier grass but not forage maize. Mixed silages of king grass and Napier grass exhibited similar species composition in UPGMA clustering, which was distant from the sole silages of the two grasses (Figure 3D).

### Microbial biomarkers in sole and mixed silages

**Figure 4:**
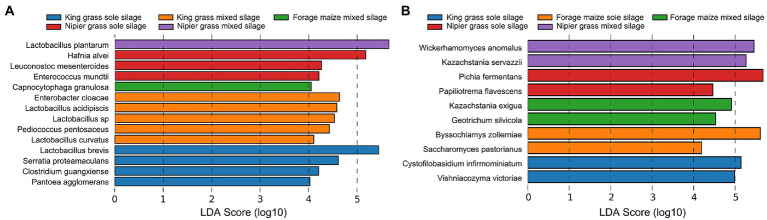
Graphics of linear discriminant analysis (LDA) effect size (LEfSe) of the biomarker prediction profiles between samples. The threshold on the logarithmic LDA score for discriminative features was set to 5.0 at an FDR-adjusted *p*-value < 0.05.

The linear discriminant analysis (LDA) effect size (LEfSe) method was used to examine the differences in microbial communities between samples and identify the bacteria and fungi in each sample (LDA score > 4.0) capped at *p*-value <0.05 (Figure 4). The biomarkers of bacterial taxa in sole silages of king grass were *L. brevis*, *S. proteamaculans*, *Clostridium guangxiense*, *Pantoea agglomerans*, and unidentified bacteria belonging to Enterobacteriales and Caloramatoraceae. In king grass mixed silage, the bacterial biomarkers were *E. cloacae*, *Lactobacillus acidipiscis*, *Lactobacillus curvatus*, and *Pediococcus pentosaceus*. The discriminatory bacterial biomarkers for the sole silage of Napier grass were *H. alvei*, *Leuconostoc mesenteroides*, and *Enterococcus mundtii*. By contrast, in Napier mixed silage, *L. plantarum* was discriminant. For biomarkers of fungal taxa, six differentially abundant species in sole silages and four in mixed silages were identified. In sole silages, *C. infirmominiatum* and *V. victoriae* were discriminant in king grass, *B. zollerniae* and *Saccharomyces pastorianus* were discriminant in forage maize, and *P. fermentans* and *Papiliotrema flavescens* were discriminant in Napier grass. In mixed silages, fungal biomarkers were identified only in forage maize and Napier grass and included *K. exigua* and *G. silvicola* in forage maize and *W. anomalus* and *K. servazzii* in Napier grass.

### Network-wide correlation analysis

**Figure 5:**
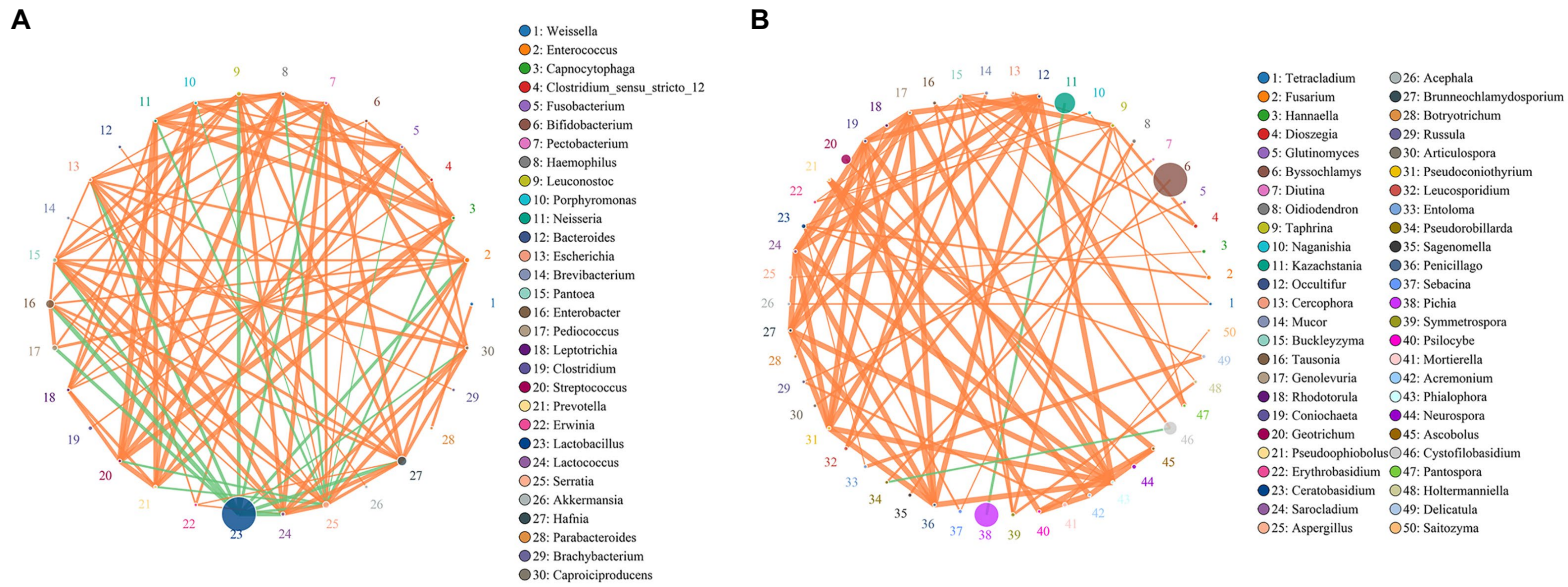
Taxa–taxa interactions at the species level in **(A)** bacterial and **(B)** fungal co-occurrence networks using Spearman’s rank correlation analysis set at a permutation value of 200, correlation >0.6 and *p*-value <0.05. Circles represents species and size of circle represents abundance. The edges represent the correlation between the two species. The thickness of the edge represents the strength of the correlation. The orange line represents a positive correlation and the green line represents a negative correlation.

The co-occurrence network is a form of correlation analysis to evaluate bacterial and fungal interactions in environmental samples. To identify the common positive and negative interactions between microbiota during fermentation, samples were combined and the correlations were calculated using Spearman’s rank correlation analysis, with a correlation value of >0.6 with 200 permutations at *p*-value <0.05 (Table 3). The taxa networks of bacteria consisted of 32 nodes with 164 positive and 18 negative correlations (Figure 5A). *Lactobacillus* was predominant in the network, showing a strong negative correlation with several bacterial species, such as *Hafnia*, *Pantoea*, *Enterococcus*, and *Enterobacter*. *Hafnia* and *Enterobacter* were relatively abundant and positively correlated with each other. Importantly, both genera showed a strong positive correlation with *Leuconostoc*, *Enterococcus*, *Serratia*, and *Lactococcus* and were negatively correlated with *Lactobacillus*. For the fungal network, which showed higher genera richness than the bacterial network, 80 nodes with two negative correlations and 76 positive correlations were identified (Figure 5B). *Byssochlamys* was enriched with a positive correlation with *Pseudorobillarda*, which showed negative correlation with the enriched fungal genus *Cystofilobasidium*. Moreover, a negative correlation was identified between *Kazachstania* and *Pichia*, which were both relatively abundant during fermentation but antagonistic in the fungal network.

**Table 3:** Topological properties of communities in bacterial and fungal networks.

Network properties	Bacterial network	Fungal network
Number of nodes	32	51
Number of edges	112	78
Modularity	0.255	0.323
Network density	0.367	0.261
Average shortest path length	2.034	1.924
Average clustering coefficient	0.742	0.605

## Discussion

This study compared the silage fermentation effects between sole silages of gramineous forages and mixed silages with forage legumes and the diversity of their microbial communities. Poaceae grasses, in this study, had relatively higher DM than legume crops in fresh materials. The DM content of the three grasses relatively decreased after 60 days of ensiling compared with that of the fresh grasses, and the content of DM in mixed silages with forage legumes was generally lower than that in sole silages. Although the decrease of DM contents may increase the possibility of spoilage—usually due to fermentation caused by bacteria, such as *Clostridium* and *Enterobacter*, and some yeasts—the rapid proliferation of LAB, such as *L. plantarum* and *L. brevis*, decreased pH to 3.85–4.05 in mixed silages and 4.05–4.21 in sole silages, which somewhat prevented aerobic spoilage. In high-DM silages, for example the sole silages of grasses in this study, epiphytic LAB has insufficient water activity, and silage fermentation could be curtailed because of a lack of metabolic moisture for lactobacilli growth (Wang et al., 2018).

Quality silage is dependent on rapid proliferation of LAB, and epiphytic LABs convert WSC into organic acids, particularly LA, under anaerobic conditions, which reduces silage pH and inhibits the growth of undesirable bacteria, consequently minimize nutrition losses. The WSC content together with the activity of epiphytic LABs determines the rate of decline in pH during the early stages of ensiling, which is important for the production of stable silages (Wang et al., 2017). Although the WSC content of forages legumes was much lower than that of grasses in our study, the silages inclusion of legumes showed higher LA contents than the sole silages. As the previous study inferred, the inclusion of legumes could promote LA fermentation, which might be related to the associated effect of microbial community from the different crops (Wang et al., 2017). The results of microbial communities diversity in this study also demonstrated that the diversity of bacterial communities in the mixed silages of king grass and Napier grass were significantly higher than that in the sole silages, while the diversity of the fungal communities in the mixed silages of the three grasses were significantly reduced, indicating that the inclusion of forages legumes may introduce dominant LAB resources and had a positive interaction with LABs in grasses, thus inhibiting the reproduction and expansion of undesirable fungus. The positive interaction of LAB in mixed silages leads to a lower pH and higher LA content than that in sole silages, which are important index to evaluate the quality of fermentation. As reported before, pH values <4.0 may inhibit the growth of undesirable bacteria like clostridial (Muck et al., 2018; Hisham et al., 2022). LA might contribute the most in reducing silage pH because it is 10 ~ 12 times stronger than other organic acids. In this study, LA concentration significantly increased in mixed silages compared with that in sole silages, which indicates that the inclusion of forages legumes contributed the increasing of LA contents, as reported in previous study in alfalfa (Wang et al., 2018). In addition, AA had the second-highest concentration among organic acids in silages, ranging from 1 to 3% DM (Kung et al., 2018). In this study, although AA concentrations in two sole silages were <1% DM, which increased to 1.25–1.4% DM in mixed silages, the ratios of LA/AA were all higher than 2:1, which represents proper fermentation (Wang et al., 2017). The increase of AA content in mixed silages may be due to the attachment of acetic acid bacteria in leguminous forages. As reported in alfalfa, acetic acid bacteria probably attach to alfalfa, which could metabolize fructose and glucose *via* phosphate pentose pathway, with acetaldehyde as an intermediate, producing acetic acid before anaerobic conditions are achieved (Wang et al., 2018).

In this study, CP in silages increased after ensiling, and mixed silages had higher CP than sole silages. Although according to early study, CP decreases after ensiling because of oxygen consumed during respiration and proteolysis at the onset of ensiling (McDonald et al., 1991), other recent study showing the similar results with us speculated that the increase in CP is due to excess protein produced by the fermentative microbial communities (Guan et al., 2020). As reported, the inclusion legume has considerably higher CP and relatively lower cell wall contents (aNDF, ADF and hemicellulose) than that of corn stover (Wang et al., 2017). Thus, the relative increased CP and decreased cell wall contents in mixed silages in this study indicated that an adequate amount of legume could be evenly applied at ensiling to enhance the nutritive quality.

Notably, bacterial and fungal communities are directly related to fermentation quality because ensiling depends on the interactions between several bacterial and fungal communities. In this study, a significant increase or decrease in α-diversity indexes and diversity differences of rarefaction curves indicated that microbial communities underwent great changes after 60 days of ensiling. In detail, the bacterial communities from silages of king grass exhibited greater diversity than those in forage maize and Napier grass in both sole and mixed silages. As observed in forage maize and Napier grass, silage of forage maize with forage legumes resulted in the recruitment of more bacterial species, whereas silage of Napier grass with forage legumes significantly decreased the abundance and diversity of bacterial communities. Although mixing with forage legumes superficially increased bacterial species, the decline in bacterial community diversity in mixed king and Napier grasses was due to an anaerobic condition that is unfavorable for bacterial growth, and low pH contributes to a decrease in bacterial diversity. It could be explained that the extra microbial treatment decreased the pH, leading to the inhibition of the growth of undesirable microbiota as well as the promotion of the growth of LAB species. And the domination of the LAB in a certain silage environment leads to the decreasing of the overall bacterial diversity. Similar findings have been reported by in soybean, *Moringa oleifera* and *Morus alba* (Ni et al., 2017; Wang C. et al., 2019; Wang Y. et al., 2019). However, for forage maize, which contains high sugar content that can be utilized as the carbon source, the increase in bacterial diversity might be attributed to the inclusion of whole corn fruit during fermentation that caused proliferation of sugar-fermenting microbes from forage legumes (Ning et al., 2018). In this study, the advantages of LAB communities were detected in mixed silages, especially in king grass and Napier grass. As in the results of the LDA analysis (Figure 4A), some undesired bacterial taxa appeared as the biomarkers in sole silages of king grass (*S. proteamaculans*, *C. guangxiense*, *P. agglomerans*) and Nipier grass (*H. alvei*), and in their respective mixed silages, most of its biomarkers are high-quality LABs, such as *L. plantarum*, *L. acidipiscis*, *L. curvatus*, *Pediococcus pentosaceus*. Previous studies have demonstrated that LAB, preferably lactobacilli, are the main bacterial strain with desirable functions expected to dominate in well-preserved forage silage, because of its ability to drive the lactic fermentation during ensiling (Ávila and Carvalho, 2020). As reported, the natural population of LAB in fresh plant materials is usually heterofermentative and low in number; however, LAB begin to predominate after establishing anaerobic conditions, with more than 50% abundance in ensiled crops (Nishino and Touno, 2005; McEniry et al., 2010; Li L. et al., 2015; Xu et al., 2019). The abundance of *Lactobacillus* sp. at >70% decreases species evenness and diversity and increases species dominance (Ni et al., 2017). Many studies have reported that the predominance of *Lactobacillus* sp. indicates high silage quality. *Lactobacillus* sp. produce LA and reduce the silage pH, thereby inhibiting undesirable spoilage bacteria. As our results of the co-occurrence network, bacteria of the genus *Lactobacillus* showed negatively interacted with such biomarkers like *Hafnia* and *Pantoea*, indicating their ability to resist the expansion of the undesirable bacteria.

A few studies on the dynamics of microbial communities in silages have reported changes in fungal communities; however, evaluating fermentative qualities is important and representative biomarkers can be used as biocontrol agents to improve silage quality and reduce the risk of fungal and mycotoxin contamination in feed. In this study, some yeast-like fungus and mycetes, represented by *B. zollerniae*, *W. anomalus*, *P. fermentans*, and *K. servazzii*, were detected in the silages. *Byssochlamys* sp. are undesirable, often heat resistant, and may produce mycotoxins in contaminated pasteurized food (Frąc et al., 2015). In this study, *Byssochlamys* was mainly observed in silages of forage maize and its abundance relatively decreased in mixed silages compared with sole silages. Yeasts are common components of the microbiota of forage crops. Various facultatively anaerobic and acid-tolerant yeasts are involved in silage fermentation (Elferink et al., 2000). Their activity is also considered undesirable. Under anaerobic conditions, yeasts ferment sugars to ethanol and CO_2_, resulting in a decrease in the sugar available for acid production and an increase in dry-matter loss during ensilage (Driehuis and van Wikselaar, 2000). However, yeasts are also sources of proteins and vitamin B-complex. Many yeast species have been shown to have promising antagonistic properties against the common filamentous fungi, including mycotoxigenic fungi that generally contaminate food and feed (Olvera-Novoa et al., 2002). For example, studies have reported that as early as 3 days after fermentation, *Wickerhamomyces* (60%) dominated the fermentation until 21 days (Rungchaiwattanakul et al., 2022). *W. anomalus* was dominant in all silages at intermediate periods of fermentation, with a small participation of other genera. Other reports have shown that a mixed culture of selected yeasts and an LAB can be used as a biocontrol agent in silage to improve silage quality, because the mixed culture of yeasts could reduce the oxygen content and compete with the fungus for nutrition and space in the silage (Niba et al., 2014). However, regarding the fungal communities in silage fermentation, the data obtained from functional profiles of fungal communities are unclear and should be further validated through metabolomics.

## Conclusion

The results of this study showed that the inclusion of legumes could somewhat improve the grass silages, as indicated by chemical composition, fermentation quality, and microbial diversity, in which the mixed silages of king grass had the relatively highest DM and CP contents as well as lowest NDF and ADF content. The diversity of bacterial and fungal communities in the mixed silages were influenced that increased the bacterial diversity in king grass and Napier grass and decreased the fungal diversity of the three grasses. The increased the relative abundance of *Lactobacillus* sp. had positive interactions with other LABs in mixed silages, which inhibited the proliferation of undesirable microbiota. These results suggested that mixing legumes to grass forages appears to be a feasible strategy to improve the silage quality.

## Data availability statement

The datasets presented in this study can be found in online repositories. The names of the repository/repositories and accession number(s) can be found at: https://www.ncbi.nlm.nih.gov/, PRJNA909075 https://www.ncbi.nlm.nih.gov/, PRJNA909078.

## Author contributions

LY, YZ, and XL designed the research. HW and JY prepared the plant samples. LY extracted the DNA and amplified the 16S and ITS full-length gene. LY and XL performed the data analysis. XL wrote the initial draft. YZ, HW, and LY revised the manuscript. All authors read and approved the final manuscript.

## Funding

This work was supported by the National Natural Science Foundation of China (grant number 32160329), National Key Research and Development Program of China (grant number 2021YFD1100206), and Sichuan Science and Technology Department Programs (grant numbers 2021YFN0132 and 2020ZHFP0110).

## Conflict of interest

JY was employed by Chengdu Ke’an Technology Co., Ltd.

The remaining authors declare that the research was conducted in the absence of any commercial or financial relationships that could be construed as a potential conflict of interest.

## Publisher’s note

All claims expressed in this article are solely those of the authors and do not necessarily represent those of their affiliated organizations, or those of the publisher, the editors and the reviewers. Any product that may be evaluated in this article, or claim that may be made by its manufacturer, is not guaranteed or endorsed by the publisher.

## Footnotes

^1^ https://github.com/PacificBiosciences/barcoding/
